# 

**Published:** 1853-06

**Authors:** 


### WESTERN JOURNAL

OF

### MEDICINE AND SURGERY.

### EDITORS.

---

**LUNSFORD P. YANDELL, M. D.**

PROFESSOR OF PHYSIOLOGY AND PATHOLOGICAL ANATOMY

IN THE UNIVERSITY OF LOUISVILLE.

AND

**THEODORE S. BELL, M.D.**

### INDEX TO VOL. XI.

Abortion, without suspicion of pregnancy........186
Abscess of Brain........343
Abscess of Liver........375
Anæethesia in Midwifery........235
Anderson's case of Prostatitis..206
Aneurism, spontaneous cure...342
Arsenic, poisoning by........527
Asthma, Trousseau on........521
Asylum, Lunatic, of Va........396
Atlas of Pathology........391

B

Baldness and Hats........530
Bartlett's Discourse........346
Beaumont, Dr., death of........553
Berzelius, autobiography of....266
Bite of Rattlesnake........950
Birth, extraordinary........362
Bladder in hernial sac........486
Blood, changes in disease........ 64
Blood, absence of fibrin........428
Booth on Cholera Infantum...384
Boyd's case of Gangrene........377
Bowels, obstruction of........ 78
Brain, abscess of........343
Brain of Daniel Webster........ 92
Burwell on Chloroform........435

C

Calculous Diseases........334, 345
Carpenter's Human Physiology........ 92, 309
Castration, self-executed........340
Catalepsy........349
Chemistry, Silliman's........221
Chest, wound of, penetrating...371
China, calculous diseases in....354
Chloroform, use of in Europe..469
Chloroform, new effect of........345
Chloroform in Midwifery........438
Chloroform for Puerperal Mania........ 36
Chloroform in Labor sanctioned by royal example........552
Cholera Infantum, quinine....384
Codliver Oil........ 68
Cooke on Opium........
Creosote in Menorrhagia........279
Crooks on Creosote........279
Croup, Ware on........45, 167

D

Death of Physicians........464
Diabetes, yeast in........357
Diseases, epidemic........424
Disease, verminous........378
Disease, affecting milk........532
Dose-book, Wythe's........ 93
Drake, eulogy on........ 95
Druggist's Receipt Book........414
Dysentery, treatment of........531
Dyspepsia........540

E

Epilepsy, posture in........534

Eyes, preservation of........267

F

Farcy, a case of........490
Fauces, gangrene of........ 377
Fergusson's Surgery........397
Fever, typhoid........379
" yellow, La Roche........367
Fibrin, absence of in blood....428
Fishback's case of Hernia....210
Friction in Diseases of Joints.. 79

G

Gadberry on the Hop........216
Gaines, resection of jaw........217
Gangrene of fauces........377
Gluge's Atlas........391
Graves, Dr., death of........513
Gregory, death of........276
Gross on excision Scapula.....419
" on Ovariotomy........ 39
" eulogy on Dr. Drake.... 95
" on Kentucky Surgery...327

H

Hall, Dr. Marshall........385, 551
Hats and Baldness........530
Health of Louisville........551
Hemorrhage from a tooth..... 63

Henry, resection of jaw........217
Hernia, strangulated .........210
Hernia, a case of in Paris......486
Hippocrates, Bartlett on........346
Histology, pathological.........391
Horner's Surgical Sketches.... 82
Horner, death of...............363
Humulus lupulus, febrifuge....216
I
Infanticide, a case of.......... 26
Inflammation, prevention.......422
Intestine, strangulation of.....538
Intestine, perforation of........535
J
Jaw, resection of...............217
Jeffries on case of Mr. Webster.252
Joints, friction in diseases of.... 79
K
Kentucky Med. Society.......272
Kentucky Surgery.............327
Kenny's case of fever..........379
Kirkes' Physiology............412
Knapp's case of Wound........371
L
Lactation and Conception......
La Roche, yellow fever.......367
Lardner's Natural Philosophy.415
Lithotomy, Gross on..........334
Lithotomy, singular nucleus...432
Liver, abscess of...............375
Locke's Valedictory...........415
Lunatic Asylum of Va.........396
M
Medical Association.......444, 549
Med. Association's Transactions.223
Medical Journal of Va........368
Medical Journal, new.......... 87
Medical Schools, changes in...550
Medical Letter from Paris....549
Medical Jonrnal, Buffalo.......552
Meningitis, Cerebro-Spinal....465
Menorrhagia, creosote in.......279
Menstruation, conception.......432
Meteorological phenomenon...275
Milk, changed by disease......532
Milk-sickness in Illinois........471
Miller on Anæsthesia..........235
" on Speculum Uteri.....241
Mineral Waters, analysis......352
Monstrosities..................269
Murder of Physician by his Patient.................... 74
N
Nashville Springs, analysis of..352
Nostrums, certificates to......276
O
Occlusion of Vagina, congenital.354
Opium in acute inflammation...281
Orfila, death and bequests of..............276, 360. 400
Orfila, biography of...........516
Ovarian Tumor, rupture of.... 75
Ovariotomy, Gross' case of.... 39
P
Paralysis of facial nerve........417
Pathology, Simon's...........496
Pereira, death of..............276
Perforation intestine...........535
Perineal Fistula...............206
Peritonitis, a case of..........535
Physiology, Kirkes'...........412
Physiology, Carpenter's.......309
Poisoning, arsenic.............527
Posture in Epilepsy...........534
Power, Prof., death of........365
Preservation of the eyes.......206
Prostatitis....................
Puerperal Mania, chloroform..368
Purging to prevent inflammation........................422
Q
Quinine in Cholera Infantum..384
" tartarised..............540
R
Rain, yellow.................275
Rattlesnake, bite of...........350
Ricord on Syphilis............411
Ricord's Letters on Syphilis... 91
Roberts on Meningitis.........465
S
Scapula, excision of...........419
Silliman's Chemistry.........221
Simon's Pathology............4 5
Smallpox, vaccination.........429
Speculum Uteri..............241
Spontaneous cure of Aneurism.342
Strangulation of Intestine.....538
Surgery, Kentucky...........327
Surgical Sketches............ 82
Surgery, Fergusson's.........397
Sutton's case of Infanticide.... 26
Syphilis, Ricord on........91, 411
" Wilson on..........508
" Substitnte for mercury in..........................266
T
Tartarized Sulphate Quinine..510
Taste, alteration of...........417

Thompson on Milk-sickness...471
Tooth, hemorrhage from....... 63
Transactions Med Societies.223,272
Tumor, ovarian, rupture of.... 75
U
University sf Louisville, History of....................... 3
V
Vaccination..................429
Vagina, occlusion of...........354
Vagina, obliteration of........487
Verminous Disease............378
Veratrum Viride..............519
Virginia Med. Journal........368
Visiting-list, Physicians'....... 93
W
Ware on Croup.............. 45
Waters, mineral, analysis......352
Webster, Daniel 's case........252
Webster, Daniel 's brain...... 92
Wellington, Duke of 's case.... 66
Wendel, Dr. B. F., death of... 94
What to observe at the bed side.389
Wible on M. Hall's Doctrines..335
Wilcox's case of Abscess of Liver.......................375
Williams' Letter from Paris...486
Wilson on Syphilis............507
Windle's case of Convulsions..378
Wound of the Chest, penetrating........................371
Y
Yandell, L. P.'s, History of University of Louisville........ 3
Yeast in Diabetes.............357
Yellow Fever, La Roche on...367

